# Targeting the Uncommon: A Case Report of Osimertinib Response in Advanced NSCLC Patient with Dual EGFR (E701fs and L702fs) Frameshift Deletions

**DOI:** 10.3390/curroncol33010055

**Published:** 2026-01-18

**Authors:** Angel Kwan Qi Wong, Saqib Raza Khan, Danial Khan Hadi, Daniel Breadner, Mark David Vincent

**Affiliations:** 1 Schulich School of Medicine and Dentistry, Western University, London, ON N6A 3K7, Canada; awong2028@meds.uwo.ca; 2 Verspeeten Family Cancer Centre, London Health Sciences Centre, London, ON N6A 5W9, Canada; saqib.khan@lhsc.on.ca (S.R.K.); danial.hadi@lhsc.on.ca (D.K.H.); daniel.breadner@lhsc.on.ca (D.B.); 3 Department of Oncology, Division of Medical Oncology, Schulich School of Medicine and Dentistry, Western University, London, ON N6A 3K7, Canada

**Keywords:** non-small cell lung cancer, EGFR mutation, uncommon mutation, osimertinib, OCELOT clinical trial

## Abstract

Non-small cell lung cancer (NSCLC) accounts for approximately 85% of lung cancers with adenocarcinoma being the most common subtype. Patients with stage IV NSCLC typically have poor prognosis. In these patients, identification of actionable genomic alterations allows for the selection of targeted therapy rather than chemotherapy or chemo-immunotherapy. EGFR mutations are a common oncogenic driver in NSCLC and are targetable by tyrosine kinase inhibitors (TKIs). However, most of the studies primarily focus on common mutations, which are exon 19 deletions (Ex19del) and exon 21 (L858R) point mutations, and there is inconsistent data on efficacy in the treatment of patients with uncommon EGFR mutations. Currently, the first-line treatment for patients with common EGFR mutations involves a third-generation TKI, typically osimertinib. This case describes a 66-year-old gentleman with two uncommon EGFR frameshift deletions (E701fs and L702fs). His tumor staging was denoted as cT3N2M1b, stage IVA. The patient demonstrated a radiological and biochemical response to osimertinib as part of the OCELOT clinical trial (supported by a grant from AstraZeneca), with evidence of tumor marker decline and radiographic improvement within two months of osimertinib treatment initiation. This response has been durable with continued radiological stability and biochemical improvement at 11 months and ongoing. This case will help guide management for patients with this uncommon EGFR mutations and contribute to the scarce literature of EGFR frameshift deletions in advanced NSCLC patients.

## Simple Summary

Lung cancer is the leading cause of cancer death, and most cases are non-small cell lung cancer types. Some patients have changes in their cancer genes (alterations) that can be treated with special (targeted) medicines instead of conventional chemotherapy. We describe a patient with a very rare type of gene change (EGFR; epidermal growth factor receptor) who responded well to an oral pill known as osimertinib. This case shows that patients with even uncommon, rare gene changes may benefit from this medication.

## 1. Introduction

Lung cancer is the leading cause of cancer-related death worldwide with an estimate of 2.2 million new lung cancer cases (11.4% of all cancers) and 1.8 million deaths (18.0% of all cancer deaths) in 2020 [1,2]. In Canada, lung cancer is both the most diagnosed cancer and cause of cancer death [3]. Non-small cell lung cancer (NSCLC) accounts for approximately 85% of lung cancers, with adenocarcinoma being the most common (50%) subtype [4]. Patients with stage IV NSCLC typically have a poor prognosis, with a 5-year survival range from 2% to 13% [5,6].

Tobacco smoking is the most important risk factor for lung cancer, with smokers being 10–30 times more likely to have lung cancer than never-smokers [7]. Age is also a risk factor, with the incidence of lung cancer increasing dramatically over 45 years [8]. Notably, up to 25% of NSCLC cases globally occur in never-smokers with this type of presentation being more prevalent in women and Asian populations [9]. This is thought to be linked to environmental risk factors, such as air pollution, occupational exposures, pathogenic germline variants (*TP53*, *ATM*, *CHEK2*, *EGFR*), or genetic ancestry [4].

Identification of molecular alterations helps guide targeted therapy in both early- and advanced-stage disease [10]. Next-generation sequencing (NGS) testing on tissue samples or via liquid biopsy (circulating tumor DNA [ctDNA]) is now recommended to identify target alterations, such as *EGFR* mutations, *ALK* fusions, *ROS1* rearrangements, *BRAF* V600E mutations, *NTRK* fusions, *ERBB2* mutations or *HER2* amplifications, *MET* exon 14 skipping mutations, *RET* translocations, *RAS* mutations, and other novel mutations [10]. In patients with stage IV NSCLC, the identification of these actionable genomic alterations (AGA) is clinically relevant for selecting patients who can be treated with targeted drugs upfront, rather than chemotherapy or chemo-immunotherapy. A recent nationwide database in the United States from 2023 revealed the prevalence of oncogenic drivers in NSCLC as follows: 35.5%, 17.8%, 2.8%, and 2.3% of *KRAS*, *EGFR*, *ERRB2*, and *BRAF* V600E mutations; and 4.3%, 1.2%, 1.1%, and 0.1% in *ALK*, *RET*, *ROS1*, and *NTRK* fusions, respectively [11,12]. Additionally, programmed death-ligand 1 (PD-L1) testing is also recommended to determine the use of anti-PD-1/PD-L1 immune checkpoint inhibitors [10].

According to the global guidelines from the American Society of Clinical Oncology (ASCO), National Comprehensive Cancer Network (NCCN), and Cancer Care Ontario (CCO), determination of treatment for patients with stage IV NSCLC is based on molecular features of tumor tissues, among other factors like histology, PD-L1 expression level, and patient factors, which include ECOG (Eastern Cooperative Oncology Group) performance status and comorbidities [13,14,15,16,17]. The standard of care is then either targeted treatment based on particular AGA or, for AGA-negative cohort, combination chemo-immunotherapy or single-agent immunotherapy [13,14,15,16,17].

**Figure 1:**
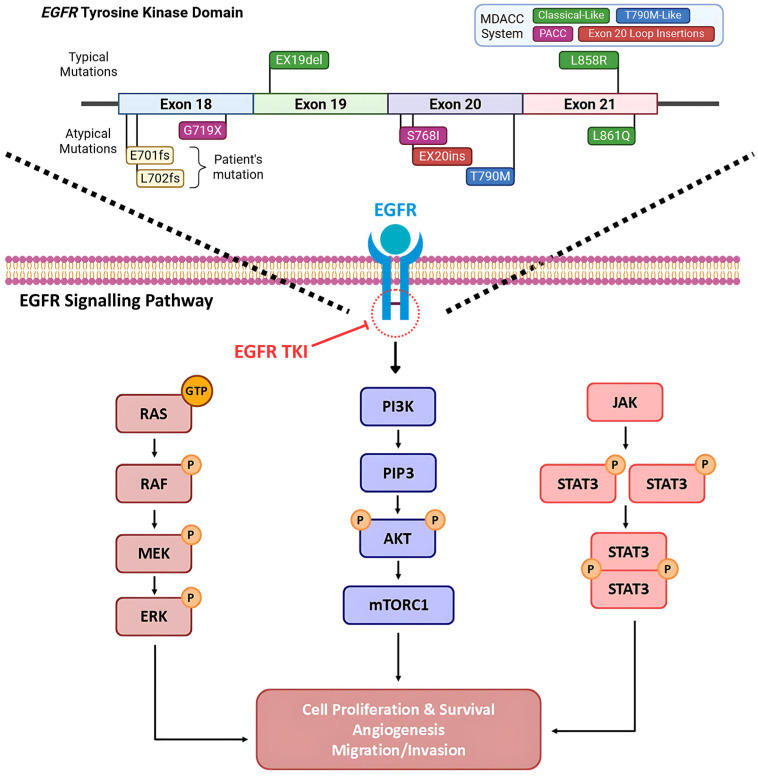
EGFR signaling pathway and our patient’s representative mutations in the EGFR TKI domain: In normal cells, the EGFR signalling pathway is activated by the binding of a ligand that causes dimerization of EGFR. This leads to downstream activation of several pathways like P13K/AKT, MAPK/RAF, and JAK/STAT. In EGFR-mutated lung cancer, activating mutations in exons 18–21 cause the EGFR kinase domain to be constitutively active. This leads to cell survival and resistance to apoptosis. MDACC: MD Anderson Cancer Center, EGFR: epidermal growth factor receptor, TKI: tyrosine kinase inhibitor, PACC: P-loop αC-helix compressing, Ex19del: exon 19 deletion, EX20ins: exon 20 insertion, fs: frameshift, ins: insertion. Arrows and perpendicular lines indicate activation/induction and inhibition/suppression, respectively.

Epidermal growth factor receptor (EGFR) is part of the tyrosine kinase type I receptor family, involved in signaling pathways that contribute to cell proliferation, survival, migration, invasion, and angiogenesis (Figure 1) [18]. EGFR mutations are traditionally classified as “typical” (common) or “atypical” (uncommon). The typical EGFR driver mutations are exon 19 deletions (Ex19del) and exon 21 (L858R) point mutations [19,20,21]. Common mutations account for approximately 85% of cases and are sensitive to osimertinib, a third-generation EGFR-tyrosine kinase inhibitor (TKI) [19,20,21]. Other mutations, such as exon 20 insertions or exon 18 point mutations, have been documented but are less common [19,22]. Recently, the MD Anderson Cancer Center (MDACC) system was established, which is an EGFR classification system based on structural analysis. Robichaux et al. examined the response to TKIs in 16,715 patients with EGFR-mutant NSCLC, identifying a structure-function relationship between mutations and drug sensitivity [22]. The groups identified were as follows: classical-like mutations that were distant from the ATP-binding pocket, T790M-like mutations in the hydrophobic core, exon 20 insertions in the loop at the C-terminal end of the αC-helix (Ex20ins-L), and mutations on the interior surface of the ATP-binding pocket or C-terminal end of the αC-helix, which were predicted to be P-loop and αC-helix compressing (PACC) (Table 1) [22]. These locations are structurally critical as this conformational compression stabilizes the active state of EGFR, leading to constitutive signaling even without ligand binding. This eventually impacts drug sensitivity and can make some tumors less sensitive to some EGFR TKI but more responsive to others. Altogether, this study indicated that uncommon EGFR mutations with a classical-like structure had a minimal effect on the overall structure of EGFR; thus, they were sensitive and selective for all classes of EGFR TKIs, both in vitro and in vivo [22].

Currently, first-line therapy for typical EGFR-driven tumors is targeted at treatment, with osimertinib being the most preferred option. Osimertinib is a third-generation EGFR-TKI that binds irreversibly to the EGFR tyrosine kinase domain, blocking downstream signaling pathways that contribute to cancer growth [14,16,18]. Osimertinib is highly active against EGFR-activating and T790M-resistant mutations [23]. Clinically, it is approved for first-line (1L) treatment of metastatic NSCLC with typical EGFR mutations and for patients who acquire the T790M mutation after earlier-generation TKIs such as afatinib (second-generation) or gefitinib/erlotinib (first-generation). The most clinical benefit has been observed in patients with common (Ex19del and exon 21 point mutations) EGFR mutations, where osimertinib in the 1L setting has demonstrated a survival benefit both with and without chemotherapy [23,24,25].

There is limited data to guide treatment for patients with rare and uncommon EGFR mutations. This study reports a 66-year-old gentleman with stage IV NSCLC who was found to have two uncommon EGFR frameshift deletions (E701fs and L702fs) and responded well to osimertinib as part of the OCELOT clinical trial. Considering these mutations are located in exon 18 and very close to E709, which is classified as a PACC mutations (Table 1), it is arguable that these could also represent PACC mutations. This case will help guide management for patients with uncommon EGFR mutations and contribute to the scarce literature of EGFR frameshift deletions in advanced NSCLC patients.

## 2. Case Presentation

**Figure 2:**
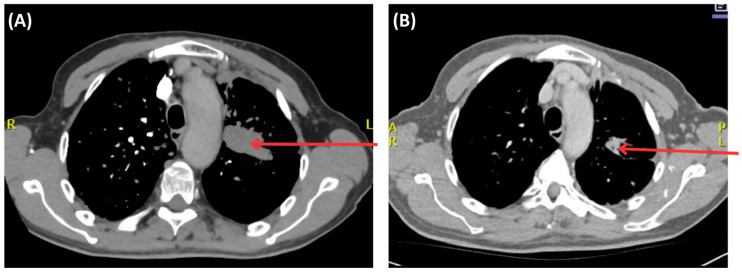
CT scan of the chest: Pre-treatment CT scan (15 August 2024) at baseline showing dominant left upper lobe/infrahilar mass [red arrow] with innumerable millimetric metastatic nodules bilaterally (**A**); most recent CT scan (5 September 2025) showing the residual focal opacity in the left upper lobe [red arrow] has decreased in size with some of the cavitary nodules being smaller, showing overall partial treatment response with osimertinib (**B**).

**Figure 3:**
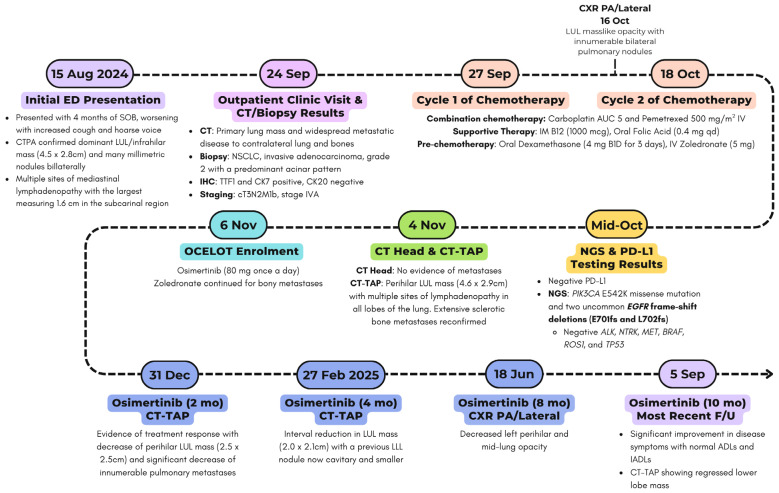
Timeline of case presentation. The timeline of this case presentation is detailed in chronological order, along with follow-up intermittent scans. ED: Emergency department, CC: Cancer Center, NSCLC: Non-small cell lung cancer, LUL: Left Upper Lobe, LLL: Left Lower Lobe, CTPA: Computed tomography pulmonary angiography, CT: Computer tomography, CXR: Chest X-ray, TAP: Thorax/Abdomen/Pelvis, IHC: Immunohistochemistry, NGS: Next-generation sequencing, AUC: area under the curve, IV: intravenous, IM: intramuscular, PD-L1: Programmed death-ligand 1, TTF1: Thyroid transcription factor 1, CK7: Cytokeratin7, mo: months, F/U: Follow-up, ADL: Activities of Daily Living, IADL: Instrumental Activities of Daily Living.

66-year-old gentleman from the Middle East initially presented to the emergency department (ED) in a tertiary care academic hospital, on 15 August 2024, with a four-month history of shortness of breath, now worsening with an increasing cough and a hoarse voice. He denied any fevers, chills, or hemoptysis. He is a lifelong non-smoker who was a professional. His past medical history is significant for gastroesophageal reflux disease (GERD), and there is no family history of malignancy. On physical examination, he looked well and rested comfortably. He was in no distress. His vital signs were within normal limits, and his chest was clear to auscultation with normal heart sounds. He underwent relevant investigations, including a chest X-ray, which was suspicious for a lung mass. On further inquiry, he mentioned that he was midway through workup for a lung mass and had a biopsy performed there before he moved to Canada. A subsequent computed tomography pulmonary angiography (CTPA) confirmed a dominant left upper lobe (LUL)/infrahilar mass measuring 4.5 × 2.8 cm and many millimetric nodules bilaterally (Figure 2). There were multiple sites of lymphadenopathy in the mediastinum, with the largest measuring 1.6 cm in the subcarinal region. Diffuse sclerotic changes were noted on the left 2nd rib and multiple levels of upper thoracic and lower cervical spine vertebrae, consistent with osseous metastasis. The patient was managed conservatively in the ED and referred to outpatient clinic for further management (Figure 3).

The patient was seen at the outpatient clinic facility on 23 September 2024. Over the prior several months, he developed a thoracic backache between the scapulae, some weight loss (estimated 20 pounds), and a cough with no hemoptysis. He admitted to mild shortness of breath on effort and a hoarse voice over the last 5 months. He reported mild anterior sternal pain, not responsive to anti-reflux therapy, and an inguinal hernia, which appeared over the last couple of months, likely related to coughing. The patient also complained of pain in the mid-humerus on the left side and some pain radiating down into the left small finger. This was likely due to vertebral involvement at the corresponding level, specifically around the C7-C8 region. On physical examination, he was alert, hemodynamically stable, and had an ECOG (Eastern Cooperative Oncology Group) performance status of one, indicating that the patient was restricted in strenuous activity but ambulatory. There was evidence of weight loss, but he was not emaciated. He has an average height of 172 cm, and he weighed 71 kg. His body mass index (BMI) was 24.0. Head and neck exams were unremarkable with no evidence of Horner syndrome or palpable lymphadenopathy. His neck vertebrae were not particularly tender, but point tenderness was identified around T2 to T3 between the scapulae. Chest and heart exams were unremarkable. The abdominal exam revealed no lymph nodes, masses, free fluid, or visceromegaly. The central nervous system (CNS) was normal with no focal or lateralizing signs. Fingers had no evidence of clubbing or nicotine staining. 

The patient provided computed tomography (CT) and biopsy results of the lung mass obtained from the Middle East. This included a CT of his head, which was unremarkable, and a CT of his abdomen and pelvis, which revealed a primary lung mass and widespread metastatic disease to the contralateral lung and bones. Histopathology confirmed a NSCLC, invasive adenocarcinoma, grade 2 (G2), with a predominant acinar pattern. Immunochemistry (IHC) revealed positive TTF1 (Thyroid transcription factor 1) and CK7 (cytokeratin7), with negative CK20. Tumor staging was denoted as cT3N2M1b, stage IVA based on clinical and radiographic findings.

As the patient was symptomatic, while waiting for the NGS testing and PD-L1 status results, he started systemic chemotherapy in September 2024 after prechemotherapy blood investigations, which were satisfactory (Table 2). He received two cycles of combination chemotherapy with carboplatin AUC (area under the curve) five and pemetrexed 500 mg/m^2^ intravenous (IV), supported with vitamin B12 (1000 mcg intramuscular), folic acid (0.4 mg orally once daily), and pre-chemotherapy dexamethasone (4 mg orally BID for three days starting day before chemotherapy) along with IV zoledronate (4 mg intravenous monthly).

Subsequently, the results of the molecular investigations from Saudi Arabia were received in October 2024, which revealed negative PD-L1 status. NGS identified a PIK3CA E542K missense mutation (VAF [variant allele frequency] 39%) and two uncommon EGFR frameshift deletions (E701fs and L702fs; VAFs of 32.8% and 21.5%, respectively). The reported EGFR VAFs represents the proportion of sequencing reads harboring each EGFR frameshift deletion, reflecting the relative abundance of each mutation within the tumor sample rather than implying two separate tumor allele. Moreover, ALK, NTRK, MET, BRAF, ROS1, and TP53 were negative.

**Figure 4:**
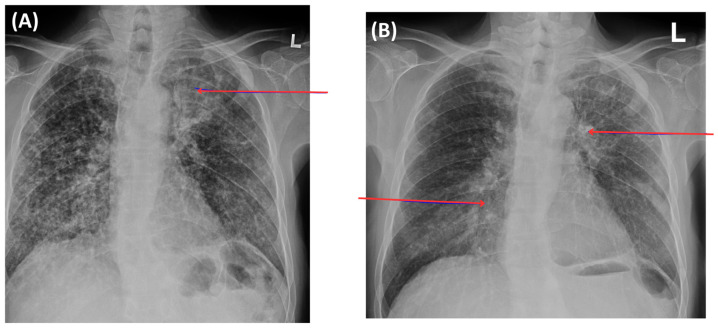
Chest X-ray (15 August 2024) at base line compared to the most recent: left upper lobe mass opacity with diffuse interstitial thickening (**A**); follow-up imaging (5 September 2025) revealed left lung opacity showing treatment response [red arrow left] and there is redemonstration and improvement in diffuse interstitial thickening [red arrow right] (**B**).

**Figure 5:**
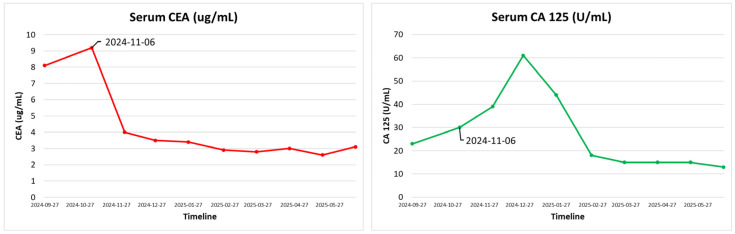
Timeline of Tumor Markers CEA, CA 125: Blood tumor markers CEA (Carcinoembryonic Antigen) and CA 125 showed notable improvement after osimertinib administration.

A multidisciplinary tumor board reviewed the case with new investigations. Given the patient’s stage IV NSCLC harboring a rare, uncommon EGFR mutation, the patient was enrolled in the OCELOT clinical trial on 6 November 2024, after obtaining informed written consent, and was switched to osimertinib 80 mg once daily, a 1L targeted therapy. OCELOT is a multicenter Canadian phase II clinical trial of osimertinib in 1L in patients with uncommon EGFR mutation (cohort B), in 3L (third-line) rechallenge of patients with EGFR-positive advanced NSCLC following 1L treatment with Osimertinib, and 2L (second-line) treatment with platinum and pemetrexed chemotherapy (cohort A) [26]. The trial is supported by a grant from AstraZeneca. The patient continued with zoledronate (4 mg IV monthly) for his bony metastases. Further CT imaging of the thorax, abdomen, and pelvis was performed on 4 November 2024, before initiating osimertinib as part of the clinical trial, which redemonstrated a left upper lobe mass (4.6 × 2.9 cm) and multiple lymph nodes, as well as bony disease, without intracranial involvement. The overall scans as per the Response Evaluation Criteria in Solid Tumors (RECIST) revealed that he did not respond well to the systemic chemotherapy and his symptoms also persisted. The patient then started to take osimertinib 80 mg daily as per the clinical trial protocol, with excellent tolerance and a significant improvement in the patient’s overall disease symptoms. He has been active and independent with normal Activities of Daily Living (ADLs) and Instrumental Activities of Daily Living (IADLs). He reported good appetite, no mucositis, no nausea or vomiting, and no diarrhea. He denied any new aches or pains. He has a minor dry cough, which is unchanged from his baseline. He denied progressive shortness of breath. His follow-up chest X-ray, taken recently, showed decreased left perihilar and mid-lung opacity, with no other significant changes (Figure 4). His blood tumor markers, specifically CEA and CA125, showed notable improvement (Figure 5), with the most recent CT scan indicating partial response as per the RECIST criteria with residual left lung opacity measuring 2.2 cm (Figure 2).

## 3. Discussion

In this report, we identified a patient with stage IV NSCLC harbouring uncommon *EGFR* frameshift deletions, E701fs and L702fs, who had a partial response to osimertinib and is currently participating in the OCELOT clinical trial. Although TKIs are effective in typical/common *EGFR* mutations, there is varied literature on their efficacy in patients with uncommon *EGFR* mutations, raising therapeutic uncertainty. Frameshift mutations specifically are extremely rare, but exon 19 frameshifts have been hypothesized to be responsive to TKI therapy, which suggest that the exon 18 frameshift mutation seen in our patient may respond similarly [27]. This case contributes to the sparse literature, specifically showcasing the effectiveness of osimertinib in these rare *EGFR* mutations.

As previously noted, in stage IV NSCLC, testing for AGA using methods such as NGS, IHC, or PCR (polymerase chain reaction) help guide therapy. Patients with an *EGFR* mutation commonly receive an EGFR TKI, which improves survival outcomes in this cohort. Current literature mainly focuses on patients with typical *EGFR* mutations, which represent around 85% of *EGFR*-mutated NSCLC. These mutations are highly sensitive to EGFR TKIs. The most clinical benefit has been observed in patients with common (Ex19del and exon 21 codon p.Leu858Arg [L858R] point mutation) *EGFR* mutations, where osimertinib in the 1L setting has demonstrated an objective response rate (ORR) of 80% with a median progression-free survival (PFS) and a median overall survival (OS) of 18.9 months (HR, 0.46; 95% CI, 0.37–0.57; *p* < 0.001) and 38.6 months (HR, 0.80; 95.05% CI, 0.64–1.00; *p* = 0.046), respectively, as per the FLAURA trial [23,24]. The FLAURA2 trial, which evaluated the addition of platinum-pemetrexed chemotherapy over osimertinib alone, showed further progression-free survival gains (median PFS, 25.5 months vs. 16.7 months; HR for disease progression or death, 0.62; 95% CI, 0.49 = 0.79; *p* < 0.001) [25]. The ORR was 83% (95% CI, 78–87%) in the osimertinib-chemotherapy group and 76% (95% CI, 70–80%) in the osimertinib-alone group [25]. The final OS data presented in World Conference on Lung Cancer (WCLC) in September 2025, reported a median OS of 47.5 months with combination therapy compared to 37.6 months with monotherapy (*p* = 0.02) [28]. However, similar to FLAURA, the FLAURA2 trial only enrolled patients with typical *EGFR* mutations.

Evidence for treatment in uncommon *EGFR* mutations mainly comes from retrospective, post hoc or pooled meta-analyses. A post hoc analysis from 2014 examined the effectiveness of gefitinib (a first-generation TKI) in patients with uncommon *EGFR* mutations (G719X or L861Q). This study found that survival time tended to be shorter among patients receiving gefitinib for such uncommon EGFR mutations compared to those receiving carboplatin-paclitaxel (11.9 vs. 22.8 months, *p* = 0.102) [29]. However, pooled post hoc LUX-Lung data showed the effectiveness of afatinib (a second-generation TKI) in patients with the G719X, L861Q, and S768I mutations. This study reported an overall survival of 19.4 months (95% CI, 16.4–26.9), objective response of 71.1% (95% CI, 54.1–84.6), and progression-free survival of 10.7 months (95% CI, 5.6–14.7) [30,31,32,33]. For the PACC subgroup of uncommon EGFR mutations, data reported an increased sensitivity with afatinib, with an ORR of approximately 56%, but with increased toxicity [22,31,34]. Hence, there is heterogeneity in the responsiveness of uncommon mutations to earlier-generation TKIs.

A multicenter, phase II trial in Korea reported the first evidence of osimertinib efficacy in metastatic or recurrent NSCLC patients with uncommon *EGFR* mutations [35]. This trial excluded typical NSCLC mutations, T790M-resistant mutations, and exon 20 insertions. Of the uncommon mutations, excluding exon 20 insertions, the study mainly included G719X, L861Q, and S768I mutations. This trial included 36 patients in the efficacy analyses and reported a median PFS of 8.0 months (95% CI, 6.8–9.2) and a median OS of 27.0 months (95% CI, 18.5–35.5) [35]. However, this study is limited by its small population size and the inclusion of both first-line treatment and additional lines of therapy. The UNICORN phase II non-randomized clinical trial also looked at the effectiveness of osimertinib in 40 patients with metastatic NSCLC and uncommon EGFR mutations, mainly G719X, S768I, and L861Q. This study excluded exon 20 insertions but included patients with common mutations if they had a coexisting uncommon EGFR mutation. This group of patients (*n* = 18) was assigned to the compound mutations arm, which also included patients with more than one uncommon EGFR mutation. The remaining (*n* = 22) had solitary mutations. They found osimertinib to be effective and reported a median PFS of 9.4 months (95% CI, 2.7–15.2). ORR was 45.5% (90% CI, 26.9–65.3) and 66.7% (90% CI, 43.7–83.7) in the solitary and compound groups, respectively [36]. Lastly, there was a phase II trial which ended early due to insufficient patient accrual. This study only included stage IV NSCLC patients with G719X, S768I, and L861Q mutations and had 17 patients enrolled. However, observational data found an ORR of 47% (95% CI, 23–72) and median PFS of 10.5 months [37]. The ongoing pan-Canadian OCELOT study of osimertinib for patients with treatment-naive advanced NSCLC with uncommon *EGFR* mutations also revealed valuable insights. Overall, the interim analysis of the study reported an ORR of 45% (95% CI, 33–58) with a disease control rate (DCR) of 77% (95% CI, 66–86) [26]. Of the 62 evaluable patients, 17 had L861X (classical-like type; ORR 8/17 = 47%), 31 had PACC (pure PACC or PACC compound mutations with other uncommon EGFR) mutations (ORR 12/31 = 38.7%), and 14 had neither (ORR 4/14 = 28.6%) [26]. There is no updated data on the uncommon co-occurring *EGFR* frameshift deletion mutation, which this gentleman has. Therefore, he is being treated with osimertinib as per the OCELOT trial, with an excellent clinical and improved radiological response.

Other TKIs have also been investigated in uncommon EGFR mutations. Cohort C of the CHRYSALIS-2 trial looked at the effectiveness of amivantamab plus lazertinib and included 105 patients with uncommon EGFR mutations (mainly G719X, L861Q, and S768I), excluding exon 20 insertions and common EGFR mutations. Amivantamab is a bispecific antibody targeting EGFR Exon 20 insertions and MET mutations, while Lazertinib is a third-generation EGFR TKI shown to have increased effectiveness against T790M-resistance mutations [38,39,40]. Of the 49 patients that were treatment naïve, the ORR was 55% (95% CI, 40–69) with a median PFS of 19.5 months (95% CI, 11.0-NE [not estimable]) [41]. Likewise, the use of lazertinib alone was also studied among 36 patients with uncommon mutations, excluding exon 20 insertions, common EGFR mutations, and the T790M-resistance mutation. The ORR was found to be 50.0% (95% CI, 34.5–65.6), and median PFS was 10.8 months (95% CI, 4.4–19.2) [42]. Specific to the PACC EGFR mutations, cohort 2 of the randomized global dose escalation FURTHER (FURMO-002) study looked at firmonertinib, a TKI approved in China for 1L advanced NSCLC for common EGFR mutations. The trial reported meaningful overall response rate in 1L setting with further investigations are still ongoing [43,44]. Anlotinib is another TKI with broad anti-tumor activity. Emerging evidence supports its use in advanced NSCLC, particularly in combination treatment approaches and in subsequent line settings following progression on platinum-based chemotherapy [45,46]. Moreover, combination of anlotinib with whole-brain radiation therapy (WBRT) appears to enhance intracranial disease control in patients with brain metastasis [47]. In our case, NGS also identified a concurrent PIK3CA mutation. Although acquired PIK3CA mutations have been suggested to contribute to resistance to TKI therapy, available evidence indicates that the presence of this concurrent mutation does not significantly impact the clinical benefit of TKI monotherapy. Further evaluation of similar cases is warranted to better clarify its impact on treatment response [48,49].

Overall, osimertinib-based therapy, either as monotherapy or, preferably, with concurrent chemotherapy, remains the first-line treatment for patients with typical EGFR mutations. Of the rare and uncommon EGFR mutations (e.g., G719X, L861Q, and S768), including PACC-like and uncommon compound PACC-like mutations, afatinib has shown improved efficacy. However, known unpleasant side effects and limited CNS penetration make this drug less attractive. Patients with an EGFR exon 20 insertion mutation may receive amivantamab (an EGFR-MET bispecific antibody) in combination with chemotherapy [50]. All other uncommon mutations are treated similarly to AGA-negative mutations and would receive platinum-doublet chemotherapy, such as carboplatin and pemetrexed, unless enrolled in a clinical trial. Our patient’s uncommon EGFR mutation falls outside the classical targets; however, due to his enrollment in the OCELOT trial, he was eligible for osimertinib and showed excellent overall improvement. Upon progression, further options for this gentleman would include palliative chemotherapy with or without immunotherapy, or new agents that target specific resistance mechanisms, as well as possible enrollment in other clinical trials.

## 4. Conclusions

The present case contributes to the rarest subset of uncommon *EGFR* mutations (E701fs and L702fs), which have shown a favourable response to osimertinib. Existing literature suggests frameshift deletion mutations to produce structural changes that may favour responsiveness to TKIs. Thus, further studies are needed to fully understand the sensitivity of these uncommon *EGFR* mutations to TKIs. Clinical trial enrolment remains critical for such patients.

## Figures and Tables

**Table 1:** Structured-based classification of EGFR mutations by the MD Anderson system [22].

Classical-Like	T790M-Like	Exon 20 Loop Insertion	PACC
L858R	**TS90M-3S**	**Ex20ins-NL**	**Primary**
Exon19dels	Classical/T790M	S768dupSVD	G719X
S720P	G719X/T790M	A767dupASV	S768I
L861Q/R	L747_K745del insATSPE	D770insNPG	L747P/S
S811F	S768I/T790M	D770del ins-GY	V769L
K754E	**T790M-3R**	**Ex20ins-FL**	E709_T710 delinsD
T725M	Ex19del/T790M/L792H	H773insNPH	**Acquired**
L833F/V	L858R/T790M/L718X	H773dupH	C797S
A763insFQEA	Classical/T790M/C797S	V774insAV	L792H
A763insLQEA		V774insPR	G724S
			L718X
			T854I

Exon20ins-NL: Exon20 near loop insertion, Exon20ins-FL: Exon20 far loop insertion, PACC: P-loop αC-helix compressing, T790M-3S: T790M-3 sensitive, T790M-3R: T790M-3 resistant.

**Table 2:** Key laboratory investigations trend at baseline, before starting osimertinib and on subsequent follow-up.

Key Laboratory Parameters	At Baseline 15 August 2024	Before Starting Osimertinib 6 November 2024	Recent Follow-Up 5 September 2025
LKC (×10^9^/L) (NR: 4.0–10.0)	11.7	7.2	8.1
ERC (×10^12^/L) (NR: 4.50–6.50)	4.74	4.45	4.67
Hemoglobin (g/L) (NR: 135–170)	135	139	126
Thrombocytes (×10^9^/L) (NR: 150–400)	276	345	203
Neutrophil (×10^9^/L) (NR: 2.0–7.5)	8.2	3.8	4.7
Lymphocyte (×10^9^/L) (NR: 1.0–4.0)	2.3	2.2	2.4
Monocyte (×10^9^/L) (NR: 0.2–0.8)	0.7	1.0	0.7
Serum CA 19–9 (U/mL) (NR: ≤34)	17	19	18
Serum CEA (ug/L) (NR: ≤5.0)	8.1	9.2	3.1
Serum CA 125 (U/mL) (NR: ≤35)	23	30	13
ALT (U/L) (NR: <51)	7	5	6
AST (U/L) (NR: <51)	12	13	12
ALP (U/L) (NR: 40–129)	263	175	77
Serum LD (U/L) (NR: ≤225)	144	181	196
Sodium (mmol/L) (NR: 135–145)	136	137	135
Potassium (mmol/L) (NR: 3.50–5.00)	4.4	4.7	4.6
Albumin (g/L) (NR: 35–52)	34	32	40
Calcium (mmol/L) (NR: 1.06–1/29)	2.05	2.08	2.19
Creatinine (umol/L) (NR: ≤130)	79	99	109

LKC: Leukocyte, ERC: Erythrocyte, CEA: Carcinoembryonic Antigen, ALT: Alanine Transaminase, AST: Aspartate Aminotransferase, ALP: Alkaline Phosphatase, LD: Lactate Dehydrogenase, NR: Normal range (reference value).

## Data Availability

The original contributions presented in this study are included in the article. Further inquiries can be directed to the corresponding author.

## Abbreviations

The following abbreviations are used in this manuscript:

NSCLC	Non-small cell lung cancer
NGS	Next-generation sequencing
ctDNA	Circulating tumor DNA
AGA	Actionable genomic alterations
PD-L1	Programmed death-ligand 1
EGFR	Epidermal growth factor receptor
ASCO	American Society of Clinical Oncology
NCCN	National Comprehensive Cancer Network
CCO	Cancer Care Ontario
ECOG	Eastern Cooperative Oncology Group
TKI	Tyrosine Kinase Inhibitor
MDACC	MD Anderson Cancer Center System
PACC	P-loop and αC-helix compressing
PFS	Progression-free survival
OS	Overall survival
ORR	Objective response rate
CI	Confidence interval
HR	Hazard ratio
DCR	Disease control rate
